# Feature diffusion reconstruction mechanism network for crop spike head detection

**DOI:** 10.3389/fpls.2024.1459515

**Published:** 2024-10-01

**Authors:** Rui Ming, Qian Gong, Chen Yang, Haibo Luo, Cancan Song, Zhiyan Zhou

**Affiliations:** ^1^ Fujian Provincial Key Laboratory of Information Processing and Intelligent Control, College of Computer and Big Data, Minjiang University, Fuzhou, China; ^2^ College of Agricultural Engineering and Food Science, Shandong University of Technology, Zibo, China; ^3^ Guangdong Laboratory for Lingnan Modern Agriculture, College of Engineering, South China Agricultural University, Guangzhou, China

**Keywords:** crop spike head, detection and counting, feature diffusion reconstruction, unmanned aerial vehicle remote sensing, precision agriculture

## Abstract

**Introduction:**

Monitoring crop spike growth using low-altitude remote sensing images is essential for precision agriculture, as it enables accurate crop health assessment and yield estimation. Despite the advancements in deep learning-based visual recognition, existing crop spike detection methods struggle to balance computational efficiency with accuracy in complex multi-scale environments, particularly on resource-constrained low-altitude remote sensing platforms.

**Methods:**

To address this gap, we propose FDRMNet, a novel feature diffusion reconstruction mechanism network designed to accurately detect crop spikes in challenging scenarios. The core innovation of FDRMNet lies in its multi-scale feature focus reconstruction and lightweight parameter-sharing detection head, which can effectively improve the computational efficiency of the model while enhancing the model's ability to perceive spike shape and texture.FDRMNet introduces a Multi-Scale Feature Focus Reconstruction module that integrates feature information across different scales and employs various convolutional kernels to capture global context effectively. Additionally, an Attention-Enhanced Feature Fusion Module is developed to improve the interaction between different feature map positions, leveraging adaptive average pooling and convolution operations to enhance the model's focus on critical features. To ensure suitability for low-altitude platforms with limited computational resources, we incorporate a Lightweight Parameter Sharing Detection Head, which reduces the model's parameter count by sharing weights across convolutional layers.

**Results:**

According to the evaluation experiments on the global wheat head detection dataset and diverse rice panicle detection dataset, FDRMNet outperforms other state-of-the-art methods with *mAP*@.5 of 94.23%, 75.13% and *R* ^2^ value of 0.969, 0.963 between predicted values and ground truth values. In addition, the model's frames per second and parameters in the two datasets are 227.27,288 and 6.8M, respectively, which maintains the top three position among all the compared algorithms.

**Discussion:**

Extensive qualitative and quantitative experiments demonstrate that FDRMNet significantly outperforms existing methods in spike detection and counting tasks, achieving higher detection accuracy with lower computational complexity.The results underscore the model's superior practicality and generalization capability in real-world applications. This research contributes a highly efficient and computationally effective solution for crop spike detection, offering substantial benefits to precision agriculture practices.

## 1 Introduction

Monitoring crop health and estimating yields are among the key challenges in precision agriculture, guiding various production stages and ensuring food security Omia et al. (2023). Crop spikes, as a crucial component of crops, represent one of the most specific manifestations of crop growth Tan et al. (2020a). They visually reflect the actual growth status of crops and are of critical importance for predicting crop yields. With the continuous development of remote sensing technology, analyzing crop-related information obtained from remote sensing platforms to predict crop growth conditions and estimate parameters has gradually become a mainstream research direction. Remote sensing-based crop spike detection can effectively achieve precise farmland management. By analyzing field information such as the density and color of crop spikes within a certain area, it can help farmers make timely decisions, assess overall yield, and forecast future harvests Zhao et al. (2021).

There are two main types of remote sensing technology: high-altitude remote sensing, represented by satellite remote sensing, and low-altitude remote sensing, primarily using unmanned aerial vehicles (UAVs) Osco et al. (2021); Rasmussen et al. (2021). Compared to satellite remote sensing, UAVs are widely used in crop growth monitoring due to their ability to capture higher-resolution remote sensing images and conduct flight operations at specific times and locations as needed Zhang et al. (2021); Sishodia et al. (2020). Currently, methods for crop spike detection based on UAV remote sensing images can be divided into two main categories: traditional image processing methods Narisetti et al. (2020); Bi et al. (2010) and deep learning-based methods Zhao et al. (2022, 2023); Tan et al. (2023). Traditional image processing techniques for crop spike detection rely on color analysis, morphological operations, and edge detection to identify and segment crop spikes. These methods analyze the visual differences between crops and the background, such as color and shape, using pixel-level operations to enhance and extract key features of crop spikes. While these techniques perform well in scenarios with lower computational resource consumption and relatively simple implementation, they may struggle to adapt to complex or dynamically changing environments and are highly dependent on parameter adjustment and initial settings.

Deep learning is mainly used to mimic the working principle of biological vision system by constructing neural network models to automatically learn key features from a large number of remote sensing images of crop spike heads, and according to the features to achieve the classification, detection and segmentation of the target, and the common methods are such as Convolutional Neural Networks (CNNs) Gu et al. (2018) and You Only Look Once (YOLO) Redmon et al. (2016); Redmon and Farhadi (2018); Wang et al. (2023). This method can automatically learn and extract high-level features from images without the need for manually setting complex parameters and rules. Compared to traditional image processing-based spike detection methods, deep learning-based methods offer higher robustness and stronger generalization capabilities. They can handle not only static image data but also dynamic video stream data, enabling real-time monitoring and prediction of crop growth processes. However, deep learning-based spike detection methods also face several challenges and limitations.

**Figure 1:**
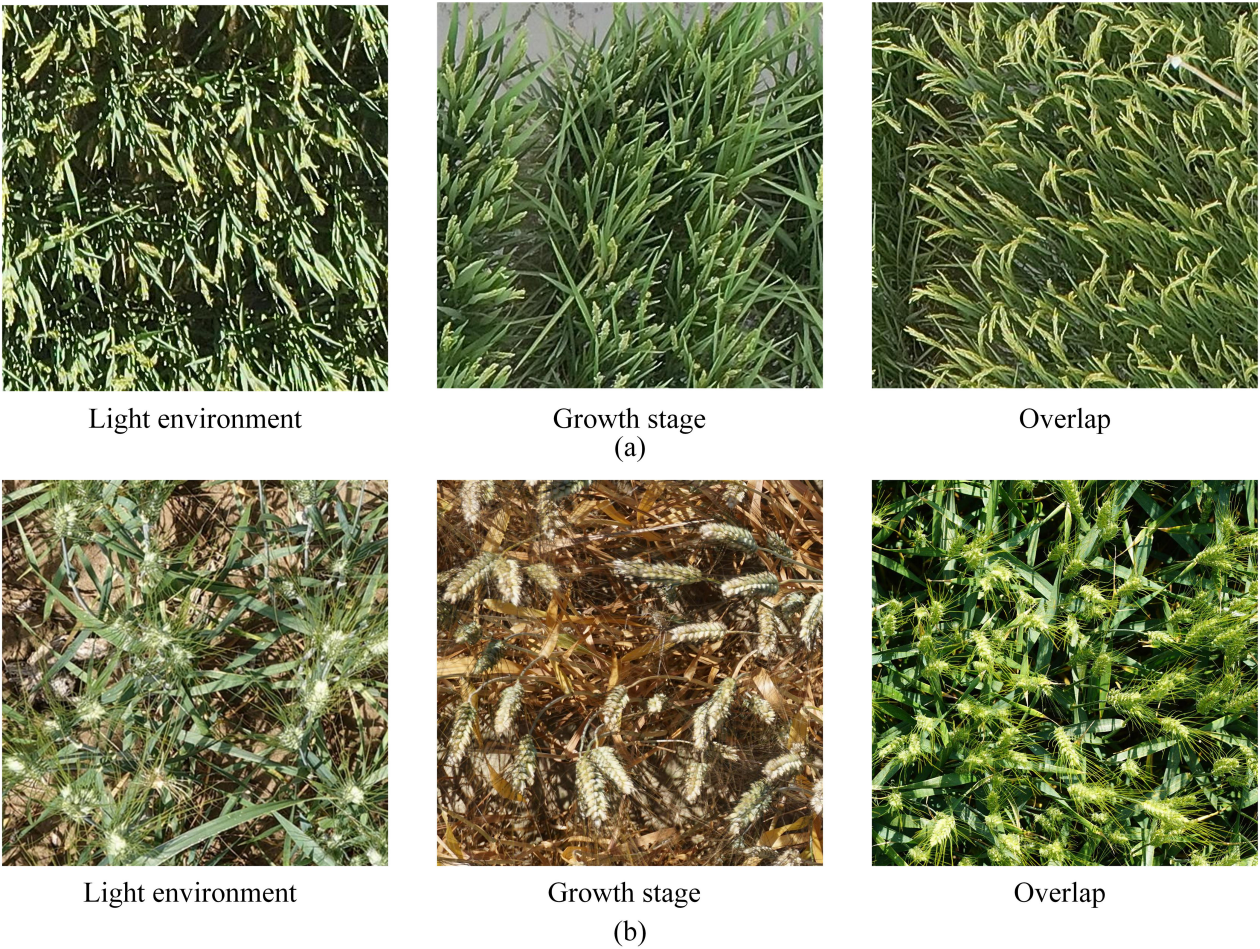
Typical states of crop spike head image related datasets: **(A)** Diverse Rice Panicle Detection; **(B)** Global Wheat Head Detection 2021.

**Figure 2:**
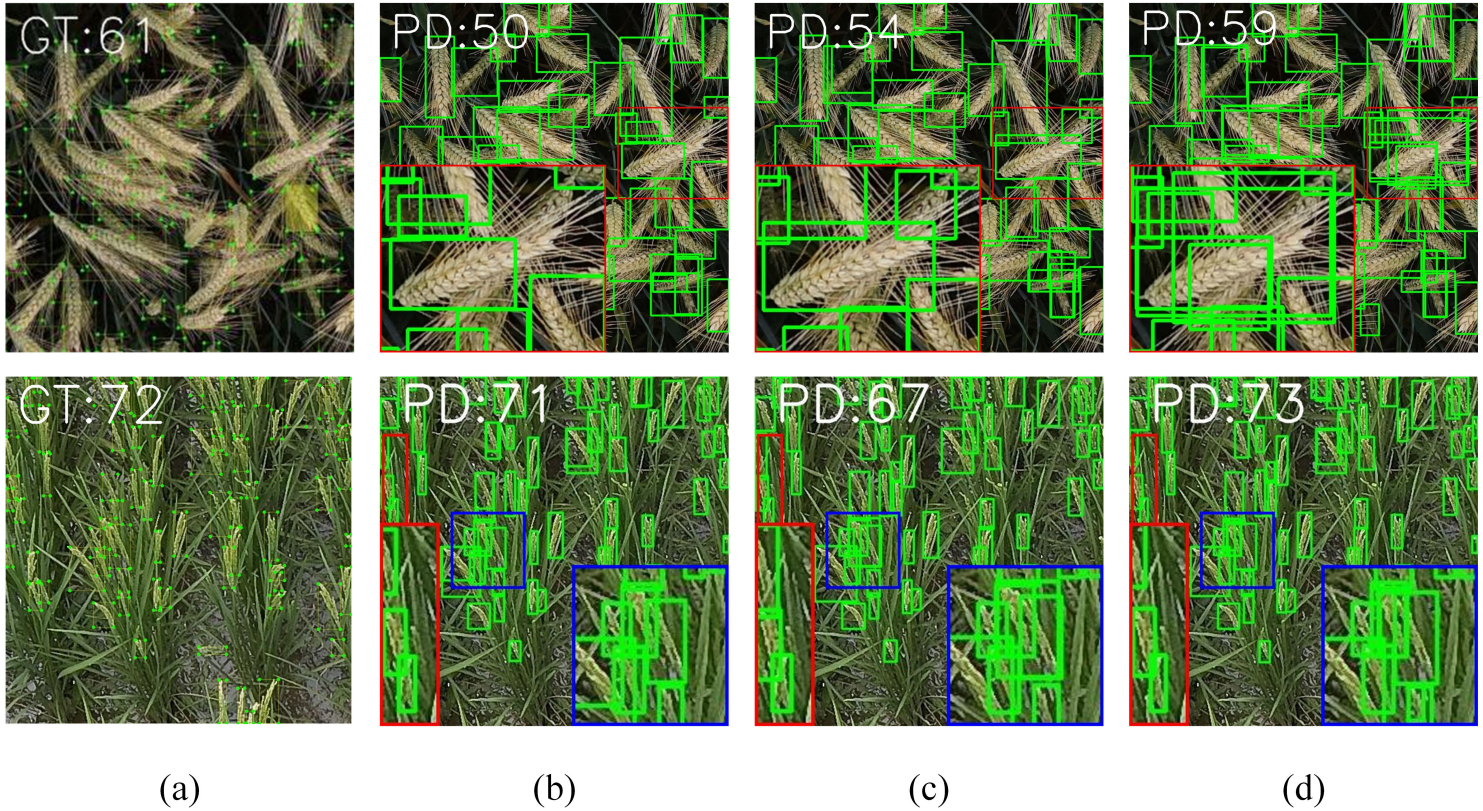
Comparison of spike head identification methods on typical datasets: **(A)** Ground Truth; **(B)** Yolov8; **(C)** WheatLFANet; **(D)** Ours.

As shown in **Figure 1**, the current stage of typical datasets related to crop spike images includes **Figure 1A**, the Diverse Rice Panicle Detection (DRPD) Teng et al. (2023) dataset proposed by Teng et al., and **Figure 1B**, the Global Wheat Head Detection 2021 (GWHD-2021) David et al. (2021) dataset proposed by David et al. From **Figure 2**, it is evident that the appearance of crop spikes changes due to varying outdoor light intensities and different growth stages, posing a significant challenge for deep learning-based spike detection methods. Additionally, when crop planting density is high, issues such as overlap and intercrossing of spikes can lead to reduced detection accuracy. Furthermore, low-altitude remote sensing images captured by UAVs are affected by factors such as image acquisition angles and flight altitudes, resulting in inconsistent scales of spikes in the remote sensing images. This inconsistency presents a challenge for deep learning-based spike detection methods. Moreover, current spike detection methods typically require substantial computational resources and storage space. However, low-altitude remote sensing platforms based on UAVs often cannot provide extensive computational resources and environments, making it crucial to achieve lightweight algorithms and improve their real-time performance.

To address the issue of reduced model recognition accuracy caused by overlapping and intersecting crop spikes, many innovative methods and techniques have been proposed. For example, Wang et al. Wang et al. (2021) introduced an image enhancement algorithm based on EfficientDet, which removes interference boxes by considering the number and size of wheat spikes in the image. This approach also incorporates an attention mechanism to improve the model’s ability to refine features. Yang et al. (2021) integrated a spatial-channel attention mechanism into YOLOv4, thereby enhancing the model’s feature extraction capability and improving recognition accuracy. Teng et al. (2023) addressed the potential feature loss of small objects in YOLOv5’s C3 blocks by proposing a Panicle-Bottleneck module, which effectively enhances the selective positioning of strong semantic and low-level features, leading to improved recognition accuracy. Furthermore, Zhou et al. (2022) enhanced the multi-scale feature extraction capability of a model by adding a Feature Pyramid Network (FPN) to the Swin-Transformer, which mitigated the recognition accuracy loss caused by overlapping and intersecting spikes. These methods demonstrate that current research primarily focuses on enhancing the model’s feature extraction capabilities through attention mechanisms and feature pyramid networks, improving the model’s robustness against overlapping and intersecting issues. However, efficient utilization of multi-scale feature information for effective crop spike detection requires further research.

These approaches often rely on high-performance computing environments, making model lightweighting a critical challenge. Various methods have been proposed for lightweighting models. For instance, Ye et al. (2023) introduced WheatLFANet, a lightweight global regression network for wheat spike detection and counting. This network compresses the input image to 1/16th of its original size using a simplified cross-stage partial spatial pyramid method in the backbone. Additionally, Khaki et al. (2022) used a truncated MobileNetV2 as a lightweight backbone feature extractor, while Bhagat et al. (2021) replaced convolutional blocks in the baseline model with Mixed Depthwise Conv, reducing the overall model parameters. These methods primarily reduce model parameters and computational complexity by optimizing the neck and backbone sections of detection models. However, since the detection head is a critical component of the model, its optimization is equally important for achieving lightweight models. The challenge lies in achieving lightweight detection heads without compromising detection accuracy, which remains an important research direction.

In conclusion, to address the aforementioned challenges, we propose FDRMNet, a novel Feature Diffusion Reconstruction Network for crop spike detection, using Yolov8 as the baseline. Specifically, we first introduce a Multiscale Feature-Focused Reconstruction(MFFR) module. This module aggregates feature information from different levels and scales, achieving comprehensive capture of crop spike information. We further process the combined features using depthwise separable convolutions with multiple kernel sizes and employ residual connections, ensuring that features at each scale possess detailed contextual information, thereby enhancing the model’s representational capacity. Building on the MFFR module, we outlining its framework separately. This network effectively captures multiscale information of crop spikes through layer-by-layer diffusion and reconstruction of features. It also enhances the perception of spike shapes and textures, allowing the network to accurately identify spikes even in complex backgrounds. As illustrated in **Figure 2**, which compares the spike detection performance of WheatLFANet, yolov8, and our proposed method on the GWHD-2021 dataset and DRPD dataset, the detection results show that all three algorithms can achieve good detection of spikes at different scales. However, yolov8 performs weakly in detecting overlapping spikes. While WheatLFANet can identify some overlapping wheat spikes to a certain extent, the recognition is not sufficiently complete.

**Figure 3:**
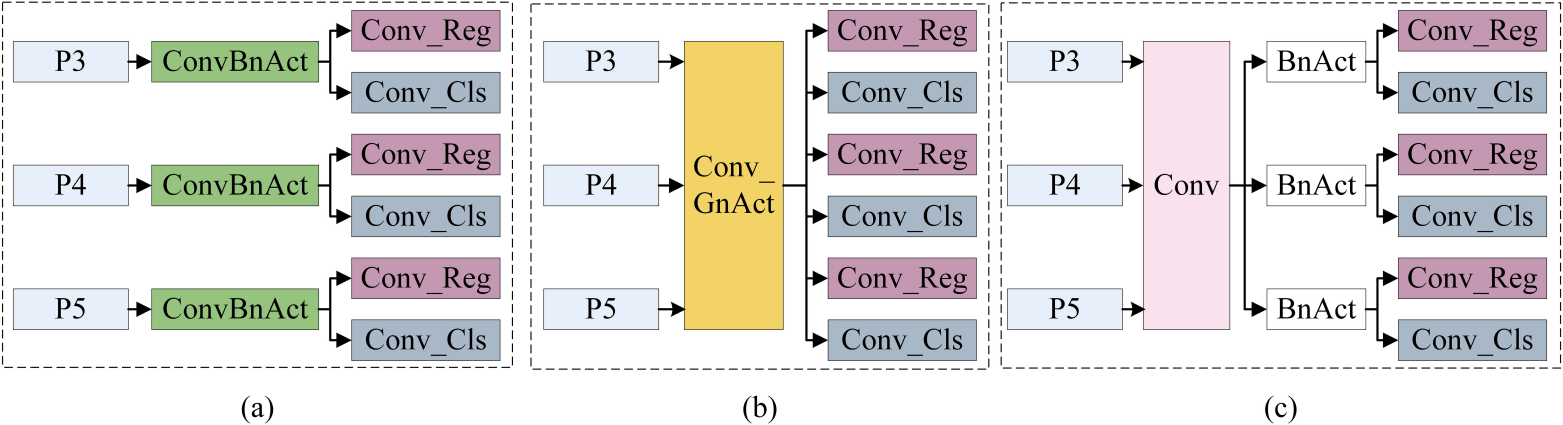
Three structures of the image detectoin head: **(A)** Independent convolutional layer and BN layer; **(B)** Shared convolutional layer and GN layer; **(C)** Shared convolutional layer and Independent BN layer.

Additionally, we introduce a Lightweight Parameter Shared Detection Head (LPSDH) to further reduce the model’s parameter count, making it suitable for the limited computational resources of low-altitude remote sensing platforms. As illustrated in **Figure 3**, the current stage image detection heads have three structural types. The first type uses independent detection heads at different feature levels, which leads to low parameter utilization efficiency since object features at relatively similar scales should be similar. The second type incorporates shared parameter detection heads into Group Normalization (GN). While this method uses shared parameters to address different feature level scales, the variability in scale features can result in decreased model performance or increased computational cost during normalization. The third type shares convolution layers in the detection head while independently computing batch normalization (BN). This approach reduces parameter redundancy by sharing convolution layers in the detection head and maintains the distinctiveness of each feature level through independent BN layers. Following the third approach, we propose LPSDH, which reduces parameter redundancy through shared convolution layers while preserving the distinctiveness of each feature level with independent BN layers. This design maintains model performance while optimizing the number of parameters and computational efficiency.

Finally, considering the real-time requirements of crop spike detection tasks in actual deployment, performance optimization was conducted. By adopting a lightweight network structure and efficient computational strategies, we optimized the C2f module and proposed an Attention-Enhanced Feature Fusion Module (AFFM). Additionally, given the substantial overlap of targets in crop spike detection, we introduced a powerful Intersection over Union with a focusing mechanism (PIoU) as the loss function.

To be concrete, our contributions are summarized as:

- We introduce a novel crop spike detection network based on a feature diffusion reconstruction mechanism network, named FDRMNet. By leveraging the MFFR module, which aggregates feature information from different levels and processes it with convolutions of varying kernel sizes, the network effectively extracts multiscale features. This enables comprehensive capture of crop spike information and enhances the perception of spike shapes and textures.
- We propose a Lightweight Parameter Shared Detection Head that reduces parameter redundancy through shared convolution layers while maintaining the distinctiveness of each feature level with independent BN layers. This optimization improves the model’s parameter efficiency and computational performance. At the same time, We optimize the C2f module by introducing an Attention-Enhanced Feature Fusion Module, which enhances the model’s feature extraction capabilities.
- Extensive experiments conducted on public datasets demonstrate that our FDRMNet outperforms state-of-the-art image fusion algorithms in both visual performance and quantitative metrics.

The subsequent sections of our paper are structured as follows: In Section II, we conduct a review of related work. In Section III, we outline the specific details of our detection method, while in Section IV, we delve into the discussion of experimental results. Lastly, in Section V, we provide the concluding remarks.

## 2 Related work

Crop spike detection is a crucial component of precision agriculture, directly impacting crop yield estimation and quality assessment. It has been a research hotspot in the field of agricultural remote sensing. In recent years, with the advancement of computer vision and deep learning technologies, image-based crop spike detection methods have made significant progress. This section briefly reviews the development of crop spike detection methods, highlighting both traditional image processing and deep learning-based approaches, and discusses the limitations of current crop spike detection methods.

### 2.1 Traditional image processing-based spike detection methods

In the early stages of crop spike detection, traditional image processing methods were primarily used. These methods detect spikes by extracting low-level features from images through morphological filtering, edge detection, and color analysis. By analyzing the visual differences between crop spikes and the background in terms of color and shape, these techniques achieve the detection and segmentation of spikes in images, enabling localization and counting Qiongyan et al. (2014). For example, Zhou et al. (2018) employed a dual-segmentation method to count wheat spikes. They first used the maximum entropy segmentation method to obtain a rough segmentation of the image, then applied morphological filters to denoise the rough segmentation results, and finally used morphological reconstruction theory to segment the adhered parts of the denoised image, achieving fine segmentation. Li et al. (2018) used a dynamic threshold segmentation method to detect wheat spikes, yielding satisfactory results.

Despite the good performance of traditional image processing methods under low computational resource consumption, they face numerous challenges in complex environments. For instance, these methods’ robustness and adaptability significantly decrease under varying lighting conditions, increased background complexity, and overlapping spikes. Traditional methods are highly dependent on parameters, often requiring meticulous parameter adjustments based on specific application scenarios, making it difficult to meet the detection needs of different crops and environments.

### 2.2 Deep learning-based spike detection methods

In recent years, deep learning has achieved significant breakthroughs in the field of computer vision, providing new solutions for spike detection methods. Deep learning-based spike detection methods primarily utilize neural networks for feature extraction and classification of images. By training on large datasets, these networks learn the feature representations of spikes, enabling automatic detection. Compared to traditional image processing methods, deep learning-based spike detection methods exhibit greater robustness and higher detection accuracy. The existing deep learning-based spike detection methods can be mainly categorized into CNNs, Region Proposal Networks (R-CNNs), and Single-Stage Detectors (SSD).

#### 2.2.1 CNN-based methods

CNNs are the most commonly used image processing models in deep learning. Through the stacking of convolutional and pooling layers, CNNs automatically extract spatial features of images. CNNs have shown excellent performance in tasks such as object detection and image classification and have been widely applied in crop spike detection. For example, Tehran et al. Sadeghi-Tehran et al. (2019) used Simple linear iterative clustering to segment images into superpixels, followed by image classification using a CNN for semantic segmentation of wheat spikes. Madec et al. (2019) applied CNN methods to segment and count wheat spikes from high-resolution images obtained via low-altitude remote sensing.

However, CNNs face challenges in real-time recognition tasks due to their complex network structures, which result in slower training and inference speeds and higher computational complexity. This makes it difficult for these models to meet the requirements of real-time recognition tasks.

#### 2.2.2 R-CNN-based methods and their variants

R-CNN and their variants represent another important class of methods in object detection tasks. These methods generate candidate regions and classify and regress these regions to achieve object localization and recognition. In crop spike detection, Faster R-CNN Ren et al. (2017) is mainly used for research, achieving object detection by generating candidate regions, extracting features, and using an R-CNN to classify and regress these regions, thereby effectively improving the inference speed of object detection. For instance, Li et al. (2022) utilized Faster R-CNN to detect and count wheat spikes, enhancing detection accuracy and speed by enhancing the region proposal network and feature extraction network. Hong et al. (2022) proposed an improved Mask R-CNN combined with Otsu preprocessing for rice spike detection and segmentation. Zhang et al. (2022) made improvements to Faster R-CNN from three aspects: the feature extraction network, scale feature maps, and Regions of Interest, resulting in significant improvements in detection accuracy according to experimental results.

Despite the excellent detection accuracy of R-CNN and its variants, their computational resource requirements remain high, especially when processing high-resolution remote sensing images. Therefore, further optimizing network structures and computational strategies to enhance real-time performance and computational efficiency is a significant challenge for the application of these methods in crop spike detection.

#### 2.2.3 Single-stage detector-based methods

Single-stage detection methods are efficient approaches that treat object detection as a single regression problem. They directly predict bounding boxes and class probabilities at the output layer, avoiding the region proposal extraction and post-processing steps of traditional methods. This method simplifies the object detection process, reduces the computational complexity of the model, and achieves fast real-time prediction results, which is suitable for applications such as crop monitoring with drones. In recent years, with the continuous development of technology, single-stage detection methods have been widely used in crop spike detection based on low-altitude remote sensing. The most commonly used single-stage detectors in the industry include the SSD and YOLO series of algorithms. A typical example of the SSD algorithm is EfficientDet Tan et al. (2020b), which is based on the EfficientNet Tan and Le (2019) backbone and uses Bi-directional Feature Pyramid Network for multi-scale feature fusion, improving model speed while maintaining high detection accuracy.

The YOLO series is particularly notable for its excellent real-time performance. YOLOv7 Wang et al. (2023) and YOLOv8, in particular, have continued to optimize the model structure and training strategies while maintaining the YOLO series’ fast detection advantage, significantly improving detection accuracy. Additionally, GOLD-Yolo Wang et al. (2024b), proposed by Wang, has incorporated an aggregation and distribution mechanism into YOLO, greatly enhancing its detection accuracy, making it one of the mainstream methods in the YOLO series today. Besides single-stage detection methods like SSD and the YOLO series, algorithms such as CornerNet Law and Deng (2018) and CenterNet Duan et al. (2019) achieve detection by focusing on keypoints of the object, further expanding the application scenarios of single-stage detection methods.

In the field of crop spike detection, there is currently a significant amount of research on single-stage detection methods. For example, Gong et al. (2020) proposed a detection method based on Yolov4 for wheat heads, improving both detection rate and speed. Additionally, Bai-yi et al. (2020) used the SSD algorithm for the first time to identify rice spikes. Although these methods have achieved some success in crop spike detection, they still face some challenges. Due to the direct regression prediction of single-stage detectors, their accuracy may be slightly lower compared to two-stage detectors. Moreover, since the size and shape of crop spikes may vary significantly between different growth stages and varieties, it is necessary to design appropriate feature extraction networks and scale transformation strategies to better adapt to the detection of targets at different scales.

To overcome these challenges, researchers have proposed many improvement solutions. For example, OSWSDet improved the YOLO framework by integrating circular smooth labels and micro-scale detection layers, enhancing the ability to detect small-sized wheat spikes and prevent detection errors Zhao et al. (2022). SpikeRetinaNet improved the detection and counting efficiency of wheat spikes by introducing weighted bi-directional feature pyramid networks, focal loss, and attention modules, and using soft non-maximum suppression to address occlusion issues Wen et al. (2022). Panicle-Cloud Teng et al. (2023) uses YOLOv5 as the baseline model, effectively enhancing its detection accuracy for rice panicles by introducing an attention mechanism and improving the model’s receptive field for small objects. Additionally, WheatLFANet Ye et al. (2023) proposed a single-stage detection network based on feature encoding-decoding. This method first encodes the image in three stages, then fuses and remaps the extracted features, and finally uses a decoder to output the predicted object classes and coordinates. This approach not only significantly improves detection accuracy but also reduces the model’s size to some extent.

Existing spike detection methods have achieved satisfactory performance in practical applications. However, they often overlook the capture, fusion, and recognition of different scale target features in overlapping environments, as well as the computational performance requirements for low-altitude remote sensing platforms. To address this issue, we have designed a crop spike detection network based on a feature diffusion reconstruction mechanism, which effectively extracts multi-scale features by summarizing feature information from different levels and processing them with different sizes of convolution kernels. Additionally, we proposed a Lightweight Parameter Sharing Detection Head(LPSDH), which reduces parameter redundancy by sharing convolutional layers, while using independent BN layers to maintain the differences in features at each level, optimizing the quantity of model parameters and computational efficiency to a certain extent.

## 3 Methodology

In this section, we first introduce the detailed architecture of our method, followed by a detailed description of the loss function.

### 3.1 Overall framework

**Figure 4:**
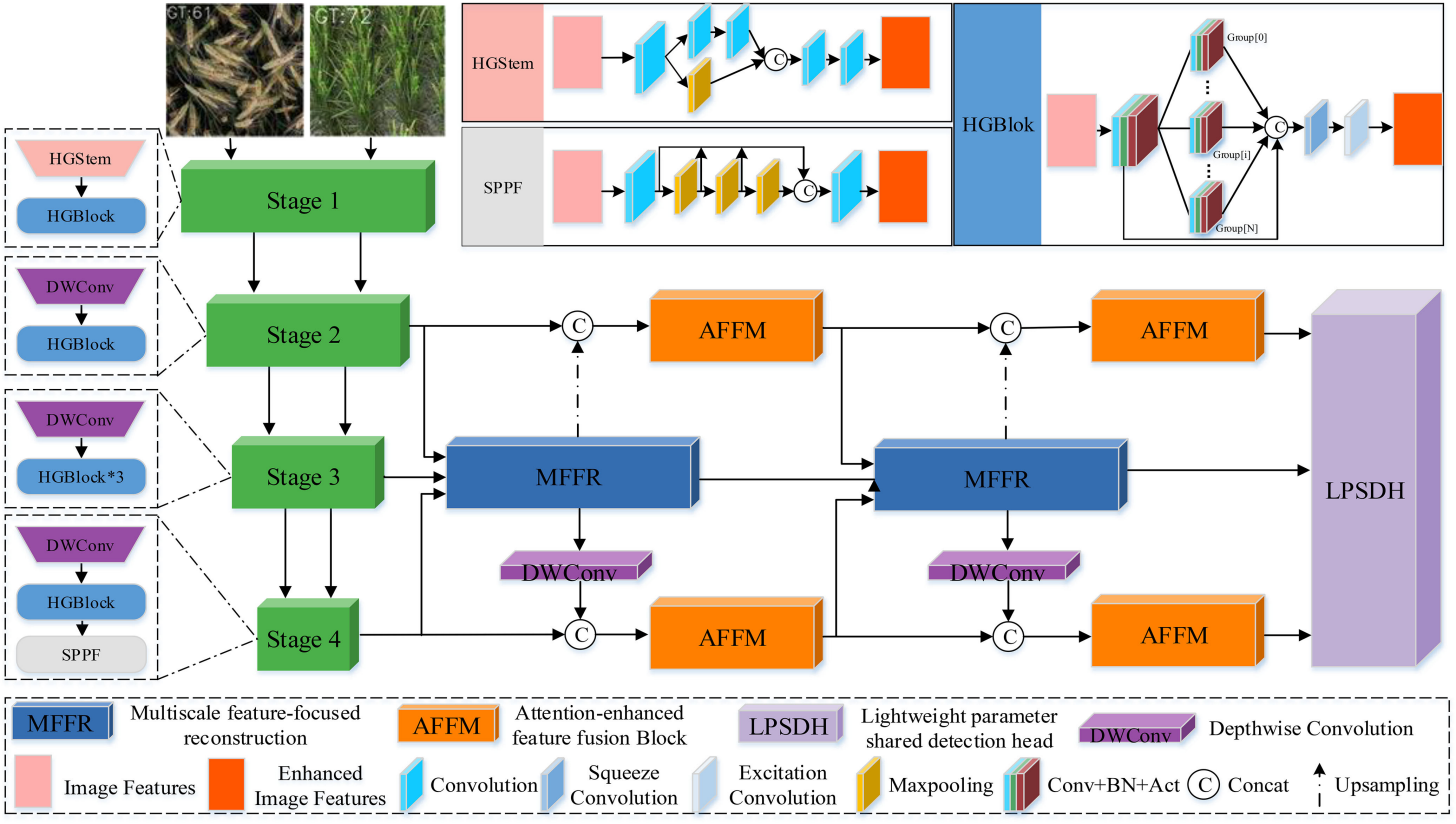
The overall architecture of the proposed FDRMNet method.

Our FDRMNet is a standard object detection framework composed of three main components: a lightweight backbone based on HGNet-v2 Zhao et al. (2024), a neck with a feature diffusion reconstruction mechanism, and a lightweight parameter-sharing detection head. The specific workflow is illustrated in **Figure 4**. Specifically, we inject the features S2, S3, S4 extracted from the last three stages of the backbone into the neck for feature fusion. The neck, equipped with the feature diffusion reconstruction mechanism, aggregates feature information from different levels and processes them with convolutional kernels of varying sizes, converting multi-scale features into image features. Finally, the lightweight parameter-sharing detection head generates the class and bounding boxes from the fused features, completing the object detection task.

### 3.2 Backbone

In existing object detection frameworks, the backbone component predominantly employs traditional convolutional layers for feature extraction. While convolutional layers excel in feature extraction from images, they may encounter issues such as vanishing gradients and computational redundancy as the network depth increases. Therefore, we have adopted HGNet-v2 as the backbone in our network, which combines an efficient network structure with lightweight components to achieve better feature extraction and computational efficiency. HGNet-v2 primarily utilizes the HGStem and HGBlock modules.

As shown in **Figure 4**, HGStem captures features at different scales by using convolutions and pooling layers of various sizes, allowing for rapid feature extraction in the early stages of the network. By reducing the spatial resolution of feature maps, it decreases computational load, thus contributing to a lightweight design. HGBlock forms the main body of the convolutional neural network using multiple ConvBNAct modules, and further improves computational efficiency and reduces model parameters through grouped convolution. Additionally, the introduction of Depthwise Separable Convolution (DWConv) Chollet (2017) further reduces the overall model parameter count and computational load. The inclusion of the SPPF module separates contextual information to minimize information loss.

Specifically, for a given image $I\in {\mathbb{R}}^{H\times W\times 3}$ to be used for object detection, we extract features at different levels through four stages:


(1) $$\text{stage}1:\;F_{vi}^{1}=HGBlock\left(HGStem\left(I_{vi}\right)\right),$$

(2) $$\text{stage}2:\;F_{vi}^{2}=HGBlock\left(DWConv\left(F_{vi}^{1}\right)\right),$$

(3) $$\text{stage}3:\;F_{vi}^{3}=HGBlock_{3}\left(HGStem\left(F_{vi}^{2}\right)\right),$$

(4) $$\text{stage}4:\;F_{vi}^{4}=SPPF\left(HGBlock\left(DWConv\left(F_{vi}^{3}\right)\right)\right),$$

where $F_{vi}^{i}$ represents the image features at the *i*-th scale in the backbone. $HGBlock_{i}$ represents the use of *i*-th HGBlock in HGNet-V2. DWConv Represents a depth-separable convolution operation

### 3.3 Neck

In the task of object detection, feature extraction and fusion are crucial, especially for images of crops in fields where multiple objects of different sizes and scales may appear in the same image. Therefore, in the neck part of the network, we propose a MFFR module for extracting and fusing features of different scales, and an Attention-enhanced Feature Fusion Module for enhancing the feature extraction capability.

#### 3.3.1 Multiscale feature-focused reconstruction module

**Figure 5:**
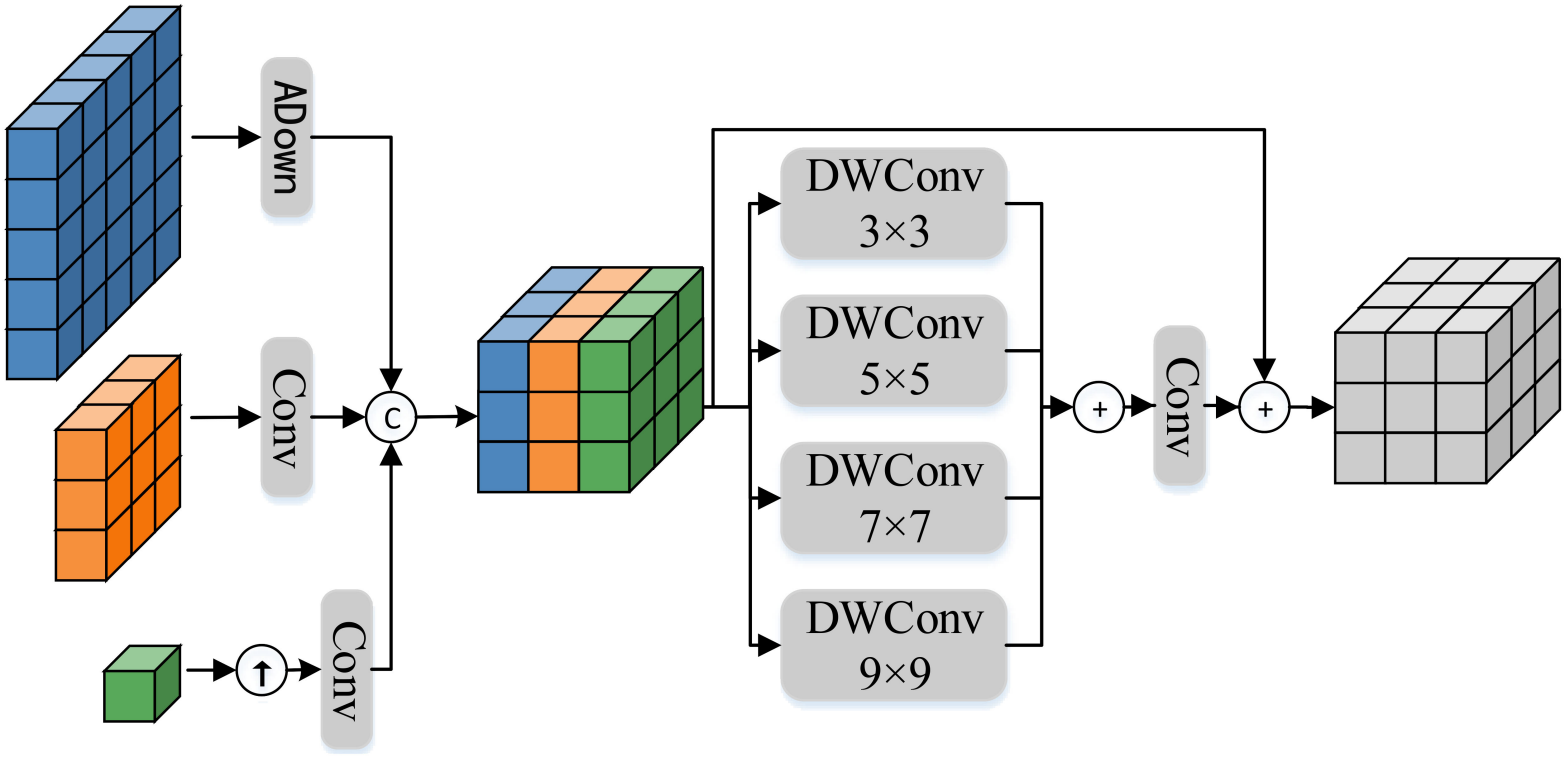
The architecture of the proposed Multiscale feature-focused reconstruction module.


The architecture of MFFR module is shown in **Figure 5**. Specifically, the input feature $\left\{F_{vi}^{2},F_{vi}^{3},F_{vi}^{4}\right\}\in R^{H^{i}\times W^{i}\times C^{i}}$ undergoes upsampling or downsampling to ensure scale consistency. Further, the aligned features are concatenated to achieve multiscale feature focus. The process of feature focus can be expressed as follows:

(5) $$F_{ff}=Concat\left(ADown\left(F_{vi}^{2}\right),Conv\left(F_{vi}^{3}\right),Conv\left(↑\left(F_{vi}^{2}\right)\right)\right),$$

where ADown is a downsampling module proposed in yolov9 Wang et al. (2024c), which can reduce the number of parameters while maintaining the detection accuracy of the target. ↓, ↑ respectively represents operations where the features undergo upsampling and downsampling.

Furthermore, by using DWConv with different kernel sizes, the focused features are processed to capture contextual information at different scales, and all contextual information is merged to generate a more comprehensive and detailed feature representation. Specifically, the feature reconstruction process utilizes DWConv with kernel sizes of 3 × 3,5 × 5,7 × 7, and 9 × 9. These four different sizes of convolutional kernels can perceive local and global information in the image to varying degrees. Moreover, based on the idea of ResNet He et al. (2016), the focused features are added to the reconstructed features, which can further improve the model’s generalization ability, ultimately achieving the functionality of the feature focusing and reconstruction module. The feature reconstruction process can be expressed as follows:


(6) $$F_{re}^{'}\;=\;Conv(\sum_{i=n}DW\;Conv_{i\times i}(F_{f\;f})),$$

(7) $$F_{re}=\;F_{ff}+F_{re}^{'},$$

where $DWConv_{i\times i}$ represents a depthwise separable convolution with a kernel size of i , and  n∈ {3, 5, 7, 9} .

#### 3.3.2 Attention-enhanced feature fusion module

**Figure 6:**
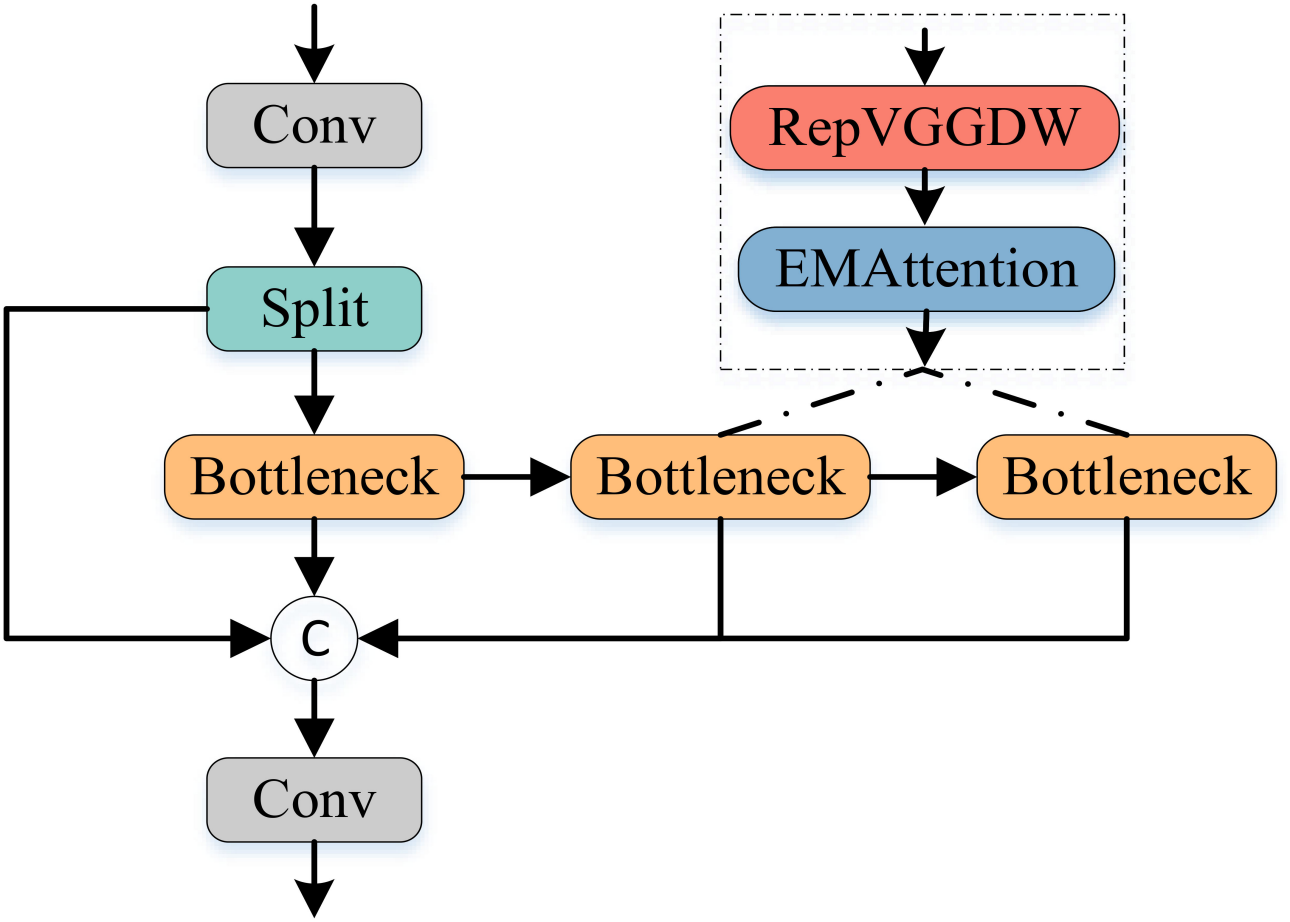
The architecture of the proposed Attention-enhanced feature fusion module.

As illustrated in **Figure 6**, we enhance the existing C2f module by incorporating the RepVGGDW block from RepViT Wang et al. (2024a) and the attention mechanism from EMANet Li et al. (2019), proposing an AFFM module. Specifically, AFFM builds upon the characteristics of the RepVGGDW block, with further optimizations applied to the token mixer and channel mixer. In the token mixer part, we introduce more complex transformations to better capture the interrelationships between different positions in the feature map. Simultaneously, in the channel mixer part, we employ additional convolutional layers to enhance information interaction between channels. This design enables AFFM to maintain efficient inference while extracting more comprehensive and detailed feature information.

Furthermore, in terms of integrating attention mechanisms, AFFM employs an adaptive approach to determine attention weights. Specifically, we aggregate channel and spatial information through adaptive average pooling and convolution operations to generate attention weights. These weights are then used to modulate the input feature map, enhancing the model’s ability to focus on important features while reducing sensitivity to irrelevant features. The forward propagation process of AFFM can be described by the following equations:


(8) $$F_{Conv}=Conv\left(F_{input}\right),$$

(9) $$F_{Split}^{1},F_{Split}^{2}=Split\left(F_{Conv}\right),$$

(10) $$F_{Bottleneck}^{1}=EM\;Attention\left(RepVGGDW\left(\left(F_{Split}^{2}\right)\right)\right),$$

(11) $$F_{Bottleneck}^{i}=Bootleneck\left(F_{Bottleneck}^{i-1}\right),$$

(12) $$F_{Output}=Conv\left(Concat\left(F_{Split}^{1},F_{Bottleneck}^{1},\cdots ,F_{Bottleneck}^{n}\right)\right),$$

where Split represents dividing the input features by 1:1, and EM Attention represents the use of the EMA attention module.

### 3.4 Head

To better address the limited computational resources of low-altitude remote sensing platforms, we employ a parameter-sharing strategy in the network’s head section. By sharing the weights of certain convolutional layers, we reduce the model’s parameter count. The architecture of LPSDH is illustrated in **Figure 3C**. Specifically, in the detection head, we design a set of shared convolutional layers that receive feature maps from different scales $\left\{P_{3},P_{4},P_{5}\right\}$ as input. Specifically, pixels are first encoded across channel context via dot convolution. Then, shared 3×3 convolutional layers aggregate channel-spatial context. Furthermore, independent BN layers are employed to maintain the distinctiveness of features at different levels. Finally, the features from each level are fed into their respective classification and regression sub-networks. The non-maximum suppression algorithm is then applied to filter out redundant detection results from the generated prediction boxes, yielding the final detection outcomes.

### 3.5 Loss function

The total loss function is combined of a Classification Loss $L_{class}$ , Bounding Box Regression Loss $L_{\text{box}}$ and a Confidence Loss $L_{\text{conf}}$ , which can be shown follows:


(13) $$L_{total}={\text{λ}}_{class}L_{class}+{\text{λ}}_{\text{box}}L_{\text{box}}+{\text{λ}}_{\text{conf}}L_{\text{conf}},$$

where $L_{class}$ , $L_{\text{box}}$ , $L_{\text{conf}}$ is a trade-off parameter, and we set to 1.0,5.0 and 1.0 respectively.

#### 3.5.1 Classification loss

$L_{class}$ is primarily used to measure the discrepancy between the detected target class and the true class. Common classification loss functions include Cross-Entropy Loss Zhang and Sabuncu (2018) and Focal Loss Lin et al. (2017). Considering that crop head images obtained from low-altitude remote sensing may exhibit multi-scale and overlapping targets, Focal Loss is preferred over Cross-Entropy Loss as it increases the loss weight of these hard-to-classify targets. This, in turn, guides the model to better learn complex features and improve detection performance. Therefore, Focal Loss is chosen as the classification loss function in this paper. It is expressed as follows:


(14) $$L_{Class}=-\alpha {\left(1-p_{t}\right)}^{\text{γ}}log\left(p_{t}\right),$$

where $p_{t}$ is the predicted probability for the true class, *α* is a balancing factor, and γ is a focusing parameter.

Furthermore, to better handle crop head images of varying complexity and reduce the impact of noisy data in real-world images on recognition performance, this paper proposes an optimization to the existing Focal Loss, called Dynamic Focal Loss. This method replaces the fixed focusing parameter γ with a dynamically adjustable parameter ${\text{γ}}_{t}$ , allowing for a smoother transition in the model’s attention to samples. The expression for Dynamic Focal Loss is as follows:


(15) $$L_{Class}=-\alpha {\left(1-p_{t}\right)}^{{\text{γ}}_{t}}log\left(p_{t}\right),$$

(16) $${\text{γ}}_{t}=\gamma_{0}+{\left(1-p_{t}\right)}^{\beta },$$

where ${\text{γ}}_{0}$ is the initial focusing parameter, set to 2.0. *β* is a parameter that adjusts the dynamic range.

#### 3.5.2 Bounding box regression loss

$L_{box}$ is primarily used to measure the difference between the position and size of the detected box and the ground truth box. The main method involves calculating the Intersection over Union(IoU) loss between the predicted box and the ground truth box. We employ the shape-IoU Zhang and Zhang (2023) to calculate the bounding box loss, which is formulated as shown below:


(17) $$L_{\text{Shape}-\text{IoU}}=1-\text{IoU}+{\text{distance}}^{shape}+0.5\times {\text{Ω}}^{shape},$$

(18) $$IoU=\frac{\left|B\cap B^{gt}\right|}{\left|B\cup B^{gt}\right|},$$

(19) $$ww=\frac{2\times {\left(w^{gt}\right)}^{scale}}{{\left(w^{gt}\right)}^{scale}+{\left(h^{gt}\right)}^{scale}},$$

(20) $$hh=\frac{2\times {\left(h^{gt}\right)}^{scale}}{{\left(w^{gt}\right)}^{scale}+{\left(h^{gt}\right)}^{scale}},$$

(21) $$distance^{shape}=hh\times {\left(x_{c}-x_{c}^{gt}\right)}^{2}/c^{2}+ww\times {\left(y_{c}-y_{c}^{gt}\right)}^{2}/c^{2},$$

(22) $${\text{Ω}}^{shape}=\sum_{t=w,h}{\left(1-e^{-\omega_{t}}\right)}^{\theta },\theta =4,$$

where *B* and $B^{gt}$ represent the predicted box and the GT box, respectively. *scale* is the scale factor, which is related to the scale of the target in the dataset, and *ww* and hh are the weight coefficients in the horizontal and vertical directions respectively, whose values are related to the shape of the GT box. $w^{gt}$ and $h^{gt}$ represents the width and height of the predicted boxes, respectively.

#### 3.5.3 Confidence loss

$L_{conf}$ is mainly used to measure the difference between the confidence that the predicted bounding box contains the target and the actual situation. We use the binary cross-entropy loss to achieve this goal, which is calculated as shown below:


(23) $$L_{\text{conf}}=\sum_{i=1}^{N}l_{i}^{\text{obj}}{\left(C_{i}-{\hat{C}}_{i}\right)}^{2}+\lambda_{\text{noobj}}\sum_{i=1}^{N}l_{i}^{\text{noobj}}{\left(C_{i}-{\hat{C}}_{i}\right)}^{2},$$

where N is the total number of prediction boxes. $C_{i}$ is the confidence level of the i-th prediction box. ${\hat{C}}_{i}$ is the true confidence of the i th prediction box, with a value of 1 when the box contains the target and 0 when the box does not contain the target. $l_{i}^{\text{obj}}$ is an indicator function with a value of 1 when the i-th prediction box contains the target and 0 otherwise. $l_{i}^{\text{noobj}}$ is an indicator function with a value of 1 when the i-th prediction box does not contain the target and 0 otherwise. ${\text{λ}}_{\text{noobj}}$ is a weight parameter used to balance the loss contribution of the prediction box with and without targets, which we set to 0.5.

## 4 Experiment validation

In this chapter, we will provide a detailed explanation of the experimental setup and implementation details of our work. Subsequently, we will present the application experiments of FDRMNet in spike detection and counting.

### 4.1 Configurations and implementation details

#### 4.1.1 Datasets and metrics

We select two widely recognized benchmarks to verify our detection performance, namely DRPD and GWHD-2021. For the DRPD dataset, the original data divides the images according to the height at which they were captured by the drone into three scales: 7 meters, 12 meters, and 20 meters. To evaluate the model’s recognition capability for multi-scale targets, we combine images from all three scales in the DRPD dataset to create a new multi-scale mixed DRPD dataset, referred to as the multi-scale DRPD (MS-DRPD) dataset. Our model is trained on the GWHD-2021 training set (2698 images) and the MS-DRPD training set (3222 images). The GWHD-2021 test set (675 pairs) and the MS-DRPD test set (537 images) are adopted to assess our detection performance.

Six objective evaluation metrics are used for comparison: precision (*Pr*), recall (*Re*), mean average precision (*mAP*), frames per second (*FPS*), parameters (*Params*), and floating point operations (*FLOPs*). Specifically: *Pr* measures the accuracy of the model, i.e., the proportion of correctly predicted targets among all predicted targets. High precision indicates fewer false positives. *Re* measures the detection capability of the model, i.e., the proportion of actual targets correctly identified by the model. High recall indicates fewer false negatives. *mAP* is the mean of the average precision (*AP*) across all classes, used to comprehensively evaluate the model’s detection performance across multiple categories and different IoU thresholds. In this paper, *mAP*@.5 is selected as the evaluation metric, representing the mAP value calculated at an IoU threshold of 0.5.*FPS* is the number of image frames the model can process per second, used to measure the model’s runtime speed. *Params* is the total number of trainable parameters in the model, used to measure the model’s complexity and size. *FLOPs* is the number of floating-point operations required for a single forward pass, used to measure the computational complexity of the model.

#### 4.1.2 Implementation details

Our experiments were conducted on a deep learning framework built on PyTorch 1.12.1+cu113 and CUDA 11.3, utilizing an NVIDIA GeForce RTX 3090 GPU (24GB) and an Intel 4310 CPU(2.10GHz).

During the training phase, the training set images were uniformly preprocessed to have a maximum side length of 640 pixels, with the width scaled proportionally. Our FDRMNet network was optimized over 200 epochs using the Adam optimizer with a batch size of 16. The initial learning rate was set to 0.01, with a final learning rate of 0.001, utilizing a multi-step learning rate decay strategy. The momentum coefficient was set at 0.937, and the weight decay at 5×10^−4^. Additionally, to prevent overfitting during the training process, we implemented an early stopping mechanism. If the model’s performance on the validation set does not improve within 50 epochs, the training will automatically stop. Notably, we did not rely on pre-trained model weights during transfer learning to ensure that our model’s performance reflects its true potential. To ensure objectivity, all comparative algorithms were implemented according to their original papers.

### 4.2 Detection comparison and analysis

In this section, we compare the detection results with state-of-the-art methods, including Yolov8, Yolov7 Wang et al. (2023), Gold Yolo Wang et al. (2024b), Faster R-CNN Ren et al. (2017), CenterNet Duan et al. (2019), EfficientDet Tan et al. (2020b), WheatLFANet Ye et al. (2023), and Panicle-Cloud Teng et al. (2023).

#### 4.2.1 Qualitative comparison and analysis

**Figure 7:**
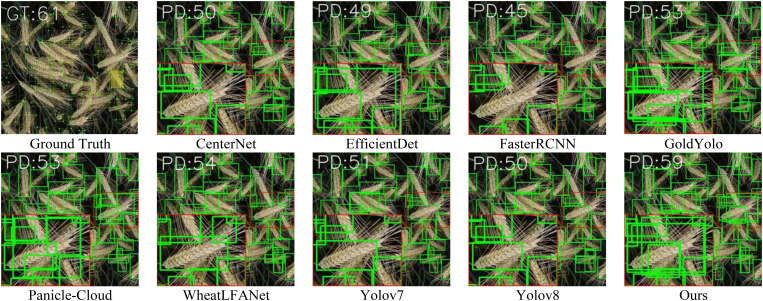
Qualitative comparison of our method with nine state-of-the-arts fusion methods on GWHD-2021 dataset.

**Figure 8:**
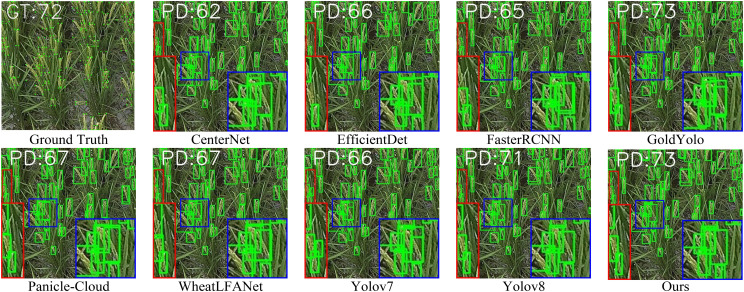
Qualitative comparison of our method with nine state-of-the-arts fusion methods on MS-DRPD dataset.

The visual results on two representative datasets, GWHD-2021 and MS-DRPD, are presented in **Figure 7** and **Figure 8**. From the recognition results in the images, it can be observed that most algorithms can accurately detect conventional spike images for the two different crops. However, there are significant differences in performance among the algorithms when dealing with complex spike images, such as those with overlaps and occlusions. As shown in **Figure 7**, which displays an overhead image of wheat at the ripening stage, the green box indicates a magnified region of the image with typical overlapping spikes. Additionally, because the overlapping spikes are at different heights, multi-scale information becomes an important factor for recognition. From the magnified region, we can see that Faster R-CNN performs poorly, failing to successfully identify multiple overlapping spikes. Although YOLOv8, YOLOv7, CenterNet, EfficientDet, and WheatLFANet are able to recognize the surface parts of spikes in the overlapping regions, they still struggle to effectively detect spikes with only partial features due to the overlap. In contrast, the algorithms with better recognition performance—Gold YOLO, Panicle-Cloud, and Ours—can accurately locate and distinguish overlapping spikes by identifying partial features, with Ours being the closest to the Ground Truth in terms of the number of spikes detected. This is primarily because our network can aggregate feature information from different levels and scales, enabling comprehensive capture and representation of crop spike information.

Furthermore, to verify the robustness of our algorithm in complex environments, we selected rice during the heading stage in an actual paddy field as the test subject, as shown in **Figure 8**. Due to the complexity of the paddy field environment and the small size and light color of rice spikes during the heading stage, the detection task becomes more challenging. The magnified region in the image highlights incomplete spike targets. From the image, it can be seen that CenterNet, Faster R-CNN, and EfficientDet fail to effectively detect spike targets, while YOLOv7, Gold YOLO, and WheatLFANet can identify the spike targets but with some false positives. In comparison, our proposed network not only identifies small targets but also effectively reduces false positives. This is mainly because FDRMNet performs multi-dimensional feature extraction on the image using convolution kernels of different sizes and enhances key features while suppressing background noise based on the attention mechanism.

Through the above qualitative experimental comparisons, it is demonstrated that our method can not only accurately detect multi-scale spikes in overlapping regions but also overcome the challenges posed by complex environments.

#### 4.2.2 Quantitative comparison and analysis

Quantitative comparison results with state-of-the-art fusion methods on the two datasets are presented in **Tables 1** and **2**. From the results, it is evident that our method achieves the best performance in terms of *Pr*, *FPS*, and *mAP*@.5. Compared to the second-best model, our method improves *Pr* by 0.7%, *mAP*@.5 by 0.45%, and *FPS* by 27 on the MS-DRPD dataset. Additionally, our model reduces the number of parameters by 50% and the GFLOPs by 24% compared to the model with the second-highest *Pr*. On the GWHD-2021 dataset, our model also shows slight improvements in *Pr* and *mAP*@.5. This advantage can be attributed to our MFFR module and the LPSDH module. The MFFR module effectively integrates feature information from different scales, enhancing the model’s adaptability to complex scenes.

**Table 1:** Quantitative comparison of our method with eight state-of-the-arts fusion methods on GWHD-2021 dataset, where boldface underlining, boldface and underline show the best, second-best values and third-best, respectively.

Method	Pr (%)	Re (%)	mAP@.5 (%)	FPS	Params (M)	GFLOPs
Yolov8	91.43	89.32	93.89	129	11.1	28.6
Yolov7	90.95	88.53	93.14	181	10.31	30.7
GoldYolo	**92.33**	**89.73**	**94.10**	**253**	13.6	29.9
Faster R-CNN	77.54	83.17	79.50	41	39.64	91.3
CenterNet	83.24	88.72	90.83	112	61.12	31.84
EfficientDet	79.83	77.38	82.19	64	**5.95**	**10.77**
WheatLFANet	90.90	84.30	90.00	164	**0.72**	**4.07**
Panicle-Cloud	92.04	**89.50**	93.98	**233**	8.14	28.6
Ours	**92.34**	88.51	**94.23**	227.27	6.80	22.7

**Table 2:** Quantitative comparison of our method with eight state-of-the-arts fusion methods on MS-DRPD dataset, where boldface underlining, boldface and underline show the best,second-best values and third- best, respectively.

Method	Pr (%)	Re (%)	mAP@.5 (%)	FPS	Params (M)	GFLOPs
Yolov8	87.07	61.54	68.19	150	11.1	28.6
Yolov7	86.39	61.41	70.29	193	10.31	30.7
Gold Yolo	**87.70**	**64.13**	**74.68**	253	13.6	29.9
Faster R-CNN	65.70	60.72	69.53	41	39.64	91.3
CenterNet	74.67	61.60	67.80	157	61.12	31.84
EfficientDet	70.73	60.64	72.25	103	**5.95**	**10.77**
WheatLFANet	86.51	61.33	68.27	164	**0.72**	**4.07**
Panicle-Cloud	87.20	61.43	70.94	**261**	8.14	28.6
Ours	**88.40**	**61.79**	**75.13**	**288**	6.80	22.7

Meanwhile, the LPSDH module reduces the number of parameters, lowers computational complexity, and thus increases detection speed.

Notably, although our method does not achieve the best performance in *Re*, the difference compared to the best method is minimal, demonstrating that our method remains competitive in terms of recall. This can be mainly attributed to our proposed feature fusion strategy, enabling the model to more accurately locate and recognize targets.

In comparison, Gold Yolo and Panicle-Cloud also perform well in terms of precision and recall but require further improvements in FPS. YOLOv8 and YOLOv7 exhibit similar performance, with higher FPS but slightly lower precision. Faster R-CNN and CenterNet, although excelling in some aspects, suffer from high computational complexity and parameter counts, affecting their practicality. EfficientDet and WheatLFANet show advantages in lightweight design but slightly lag in precision.

Additionally, as seen in **Tables 1** and **2**, there is a significant difference in recall rates between the two datasets, which is primarily due to the distinct characteristics of these datasets. The GWHD-2021 dataset consists mainly of high-resolution images of wheat spikes, with more uniform image backgrounds and clearer spike features, resulting in a higher recall rate. In contrast, the MS-DRPD dataset comprises images of rice spikes at various stages collected from actual paddy fields using drones at three different heights: 7m, 12m, and 20m. The overall background of this dataset is more complex, and the image quality is lower, leading to a relatively lower recall rate.

Overall, our method maintains high precision while achieving high detection speed, with advantages in parameter count and computational complexity.

### 4.3 Spike head counting comparison and analysis

Crop spike head counting is a primary downstream task of spike head detection and can significantly enhance the accuracy of crop parameter predictions. To validate the effectiveness of our FDRMNet network in the task of spike head counting, we randomly selected 200 images from both the MS-DRPD and GWHD-2021 datasets to evaluate the performance of our network in counting wheat heads.

In this experiment, we primarily performed linear regression on the Ground Truth (GT) and predicted values of spike heads in the images. We used the coefficient of determination (R^2^), Root Mean Square Error (RMSE), Mean Absolute Percentage Error (MAPE), and p-value as evaluation metrics for the counting results.

#### 4.3.1 Spike head counting experiment on GWHD dataset

**Figure 9:**
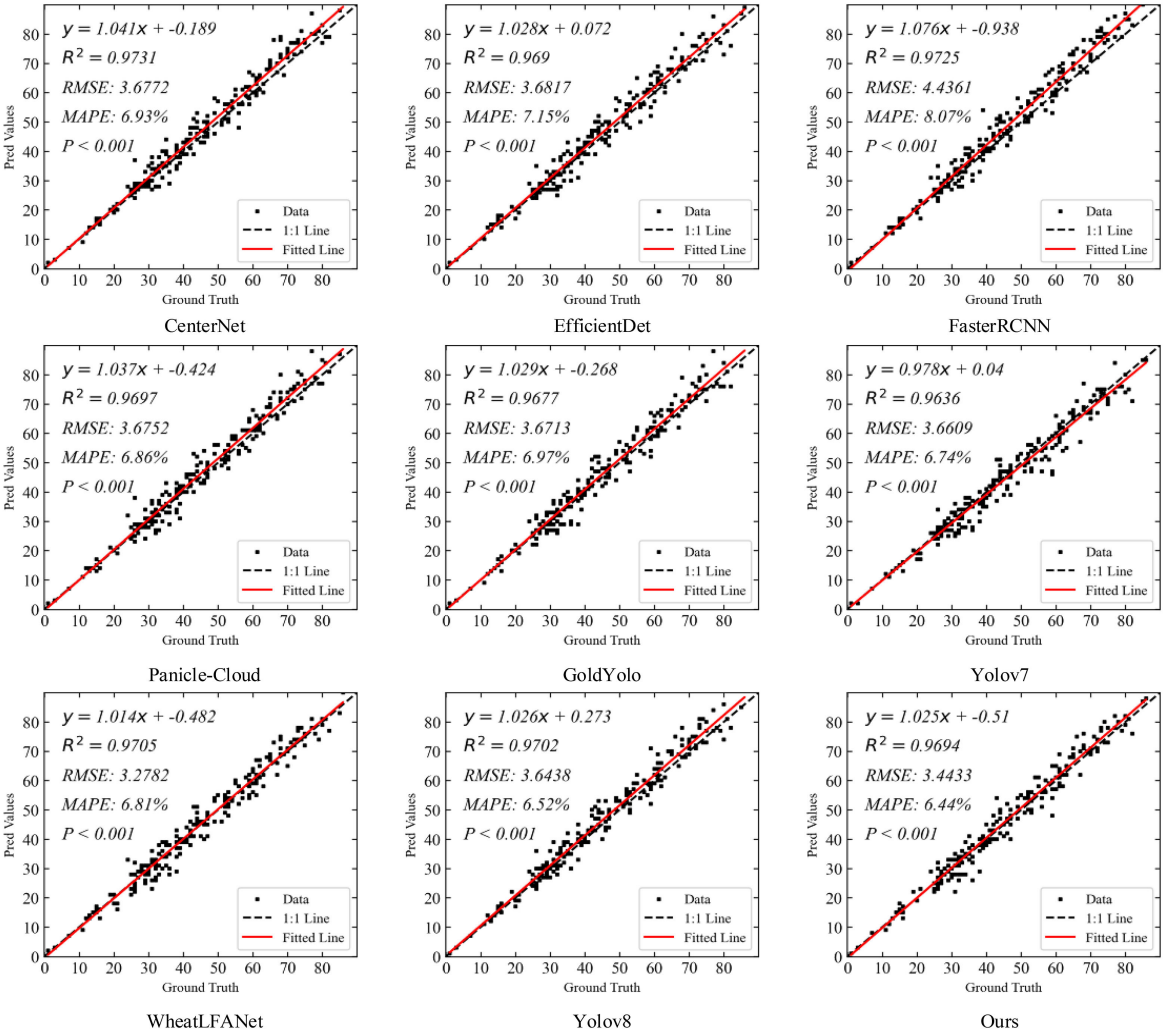
Spike head prediction correlation comparison of our method with eight state-of-the-arts detection methods on GWHD-2021 dataset.

**Figure 9** shows the spike head prediction correlation comparison of our method with eight state-of-the-art detection methods on the GWHD-2021 dataset. The results indicate that in terms of R^2^, CenterNet demonstrated the highest value of 0.9731, showcasing its strong correlation in explaining data variation. Faster R-CNN followed closely with an R^2^ of 0.9725. Our method achieved an R^2^ of 0.9694, which is also highly excellent and slightly lower than YOLOv8 (0.9702) and WheatLFANet (0.9705), indicating a high precision in capturing data variation.

In terms of RMSE, WheatLFANet showed the lowest value of 3.2782, indicating high prediction accuracy. Our method had an RMSE of 3.4433, which is only second to WheatLFANet, demonstrating similarly excellent performance. YOLOv8 and CenterNet had RMSE values of 3.6438 and 3.6772, respectively, which, although slightly higher than our method, remain within a low error range. Faster R-CNN had the highest RMSE of 4.4361, indicating relatively higher prediction errors.

Regarding MAPE, our method performed outstandingly with the lowest value of 6.44%, indicating the smallest relative prediction error and highest reliability. YOLOv8 followed with a MAPE of 6.52%, showing relatively low prediction error. In contrast, Gold YOLO had a MAPE of 6.97%, and Faster R-CNN had the highest MAPE of 8.07%, indicating relatively higher prediction errors.

Overall, our method achieves high correlation while maintaining low prediction errors, particularly excelling in relative error reduction. These results demonstrate the superiority and practicality of our method in the spike head prediction task. Although it is slightly inferior to CenterNet and Faster R-CNN in terms of R², the performance in RMSE and MAPE compensates for this shortcoming, especially with the lowest MAPE (6.44%), highlighting the significant advantage of our method in reducing relative error.

#### 4.3.2 Spike head counting experiment on MS-DRPD dataset

**Figure 10:**
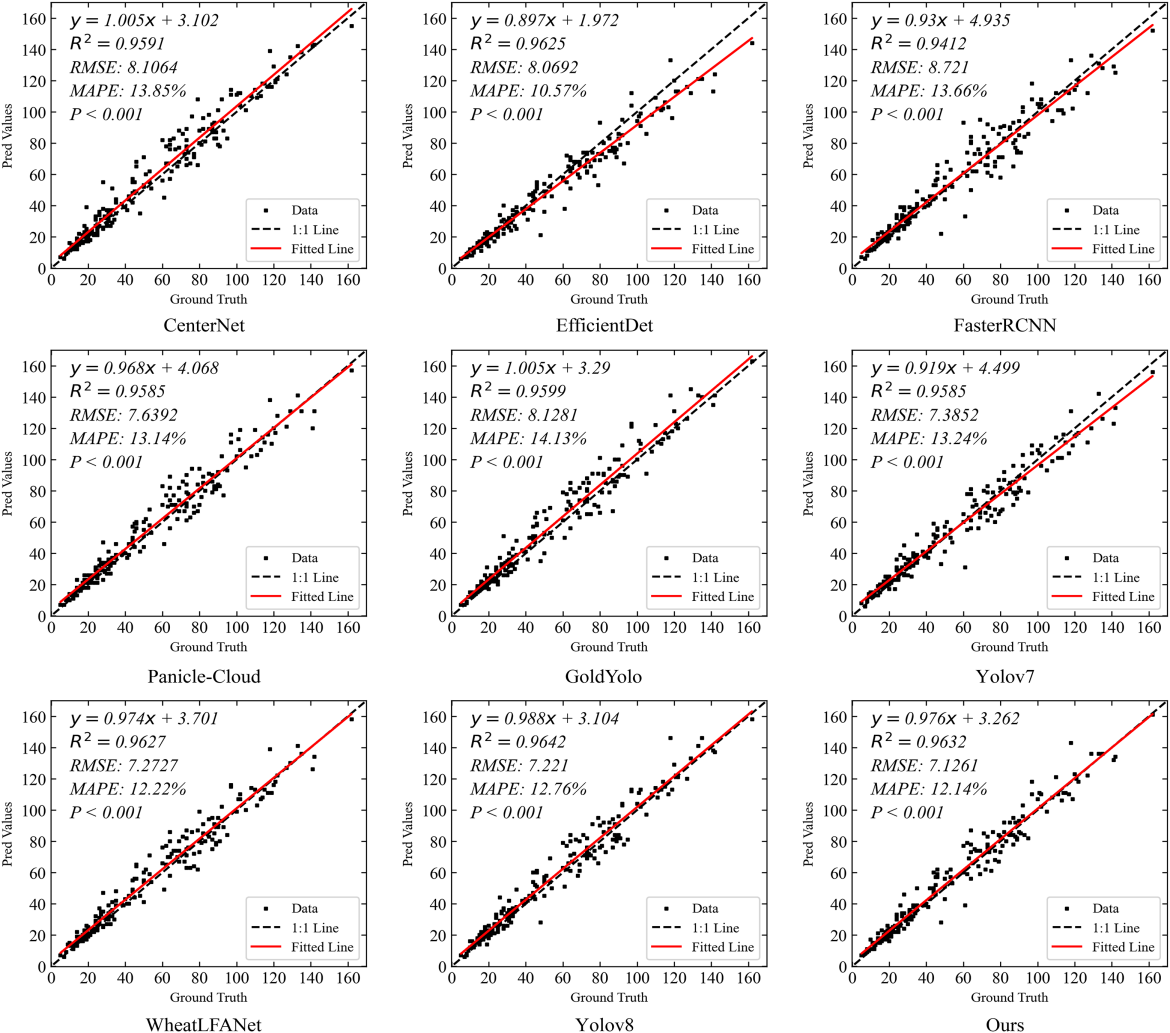
Spike head prediction correlation comparison of our method with eight state-of-the-arts detection methods on MS-DRPD dataset.

**Figure 10** shows the spike head prediction correlation comparison of our method with eight state-of-the-art detection methods on the MS-DRPD dataset. The results indicate that YOLOv8 and our method both demonstrate excellent performance in terms of R^2^, with values of 0.9642 and 0.9632, respectively. This signifies their high correlation in explaining data variation. Higher R^2^ values indicate better model fitting, thus both methods lead in prediction accuracy compared to others.

In terms of RMSE, our method achieves the lowest value of 7.1261, indicating high precision in predictions. Although YOLOv8 follows closely with an RMSE of 7.221, it remains at a relatively low level, indicating minimal prediction errors. WheatLFANet also performs well with an RMSE of 7.2727.

Conversely, Faster R-CNN shows the highest RMSE of 8.721, indicating significant prediction errors and the poorest performance among all models.

Regarding MAPE, EfficientDet exhibits the best performance with a value of 10.57%, indicating the smallest relative prediction error and high reliability. Our method also performs excellently with a MAPE of 12.14%, second only to EfficientDet. YOLOv8, with an updated MAPE of 12.76%, shows slightly higher error compared to our method but remains within an acceptable range. In contrast, Gold YOLO has the highest MAPE of 14.13%, indicating relatively larger prediction errors.

Overall, our method stands out in multiple key metrics, particularly in terms of low RMSE and MAPE, indicating high prediction accuracy and stability. While YOLOv8 performs similarly to our method in terms of R², its slightly higher RMSE and MAPE suggest that our method has an advantage in overall performance. Other methods, such as EfficientDet, show good performance in MAPE but are slightly less effective in RMSE. Thus, our method demonstrates the best overall performance, especially in minimizing errors and enhancing prediction accuracy, proving its superiority and practicality in the task of spike head prediction.

### 4.4 Ablation studies

#### 4.4.1 Multiscale feature-focused reconstruction module

We employ the MFFR module to achieve multi-scale feature extraction and fusion. Specifically, MFFR module captures contextual information at different scales by using DWConv kernels of various sizes, ultimately generating more comprehensive and detailed feature representations.

**Tables 3** and **4** present the ablation experiments for each module on the GWHD-2021 dataset and MS-DRPD dataset, respectively. From the experimental results, we observe that on the GWHD-2021 dataset, without the MFFR module, the detection Pr is 92.97%, Re is 89.88%, and mAP@0.5 reaches 94.41%. On the MS-DRPD dataset, the detection Pr is 88.33%, Re is 61.98%, and mAP@0.5 is 71.60%. These results indicate that the proposed MFFR module is highly effective in feature extraction and fusion, especially in complex scenarios.

**Table 3:** The detection performance of ablation studies on GWHD-2021 dataset, where boldface and underline show the best and second-best values, respectively.

	Pr (%)	Re (%)	mAP@.5 (%)	FPS	Params (M)	GFLOPs
w/o MFFR	**92.97**	**89.88**	**94.41**	137	10.46	32.5
w/o AFFM	91.73	89.32	94.14	**625**	7.85	**21.6**
w/o LPSDH	91.59	88.66	94.02	500	8.99	26.6
FDRMNet	92.34	88.51	94.23	227.27	**6.80**	22.7

**Table 4:** The detection performance of ablation studies on MS-DRPD dataset, where boldface and underline show the best and second-best values, respectively.

	Pr (%)	Re (%)	mAP@.5 (%)	FPS	Params (M)	GFLOPs
w/o MFFR	88.33	61.98	71.60	135	10.46	32.3
w/o AFFM	87.53	65.61	**76.06**	**625**	7.85	**21.6**
w/o LPSDH	86.90	**66.64**	75.40	500	8.99	25.8
FDRMNet	**88.40**	61.79	75.13	288	**6.80**	22.7

#### 4.4.2 Attention-enhanced feature fusion module

We utilize the Attention-Enhanced Feature Fusion Module to improve feature extraction capabilities. Specifically, AFFM enhances the comprehensive representation of features by incorporating attention mechanisms and optimizing feature fusion methods.

From the experimental results, we observe that on the GWHD-2021 dataset, without the AFFM module, the detection Pr is 91.73%, Re is 89.32%, and mAP@0.5 reaches 94.14%. On the MS-DRPD dataset, the detection Pr is 87.53%, Re is 65.61%, and mAP@0.5 is 76.06%. These results demonstrate that the proposed AFFM module is highly effective in feature extraction and fusion, significantly enhancing detection performance, especially in complex scenarios.

#### 4.4.3 Lightweight parameter shared detection head

We employ the Low-Parameter Shared Detection Head to reduce the model’s parameter count and computational load. Specifically, LPSDH achieves efficient feature aggregation by sharing weights across certain convolutional layers.

From the experimental results, we observe that on the GWHD-2021 dataset, without the LPSDH module, the detection Pr is 91.59%, Re is 88.66%, and mAP@0.5 is 94.02%. On the MS-DRPD dataset, the detection Pr is 86.90%, Re is 66.64%, and mAP@0.5 is 75.40%. These results indicate that the proposed LPSDH module maintains high detection performance while significantly reducing the usage of computational resources, making it particularly suitable for low-resource environments.

## 5 Conclusion

In conclusion, this paper proposes a novel feature diffusion reconstruction network for crop spike detection, named FDRMNet. In this network, we first design a MFFR module and its framework. This framework integrates feature information from different levels and then employs convolutional kernels of various sizes to capture global multi-scale information. Subsequently, to achieve better extraction and computational efficiency, we adopt HGNet-v2 as the feature extraction network, which combines an efficient network structure with lightweight components. Furthermore, to better capture the interactions between different positions in the feature maps, we propose an AFFM. This module utilizes adaptive average pooling and convolution operations to aggregate channel and spatial information, enhancing the model’s focus on important features. Lastly, to address the limited computational resources of low-altitude remote sensing platforms, we introduce a LPSDH. By sharing the weights of certain convolutional layers, we reduce the model’s parameter count. Our method performs excellently in spike detection and counting tasks, enhancing detection accuracy while further reducing the model’s parameter count and computational complexity. Qualitative and quantitative experiments validate the superiority of FDRMNet in terms of detection performance and metrics. Spike counting experiments demonstrate that FDRMNet offers better practicality and generalization capability in real-world applications.

For future work, we plan to explore the application of more advanced deep learning algorithms in crop spike detection, such as diffusion models, mixture of experts models, and large agricultural models, to improve the accuracy and efficiency of wheat head detection. Additionally, we will further investigate methods to enhance model lightweighting, such as introducing knowledge distillation techniques to transfer the knowledge of large, complex models to lightweight models, thereby reducing computational resource consumption while maintaining detection accuracy. We will also explore model compression techniques, such as pruning and quantization, to reduce the model’s storage requirements and increase inference speed. Lastly, we will consider multi-modal data fusion techniques by combining remote sensing images, satellite data, and ground sensor information to improve the robustness and accuracy of detection results. By integrating data from different sources, we can better address the challenges posed by complex and variable agricultural environments and enhance the model’s generalization capability. These research efforts will provide stronger technical support for the development of smart agriculture, driving crop monitoring and management towards greater intelligence and precision.

## Data Availability

The raw data supporting the conclusions of this article will be made available by the authors, without undue reservation.
